# IGF-1 Deficiency Rescue and Intracellular Calcium Blockade Improves Survival and Corresponding Mechanisms in a Mouse Model of Acute Kidney Injury

**DOI:** 10.3390/ijms21114095

**Published:** 2020-06-08

**Authors:** Samiksha Wasnik, Xiaolei Tang, Hongzheng Bi, Amir Abdipour, Edmundo E. Carreon, Brian Sutjiadi, Justin Lyu, Jintao Zhang, Sean Wilson, David J. Baylink

**Affiliations:** 1 Division of Regenerative Medicine, Department of Medicine, Loma Linda University, Loma, CA 92354, USA; swasnik@llu.edu (S.W.); xiaolei.tang@liu.edu (X.T.); hongzhengbi@zzu.cn (H.B.); Eddycarrber@gmail.com (E.E.C.); bsutjiadi@llu.edu (B.S.); jlyu@llu.edu (J.L.); jtzhang@zzu.edu.cn (J.Z.); 2 Department of Veterinary Biomedical Sciences, College of Veterinary Medicine, Long Island University, Brookville, NY 11548, USA; 3 School of Basic Medicine, Zhengzhou University, Zhengzhou 450051, China; 4 Division of Nephrology, Loma Linda University Medical Center, Loma Linda, CA 92354, USA; AAbdipou@llu.edu; 5 Henan Institute of Medical and Pharmaceutical Sciences, Zhengzhou University, Zhengzhou 450052, China; 6 The Lawrence D. Longo, MD Center for Perinatal Biology, Department of Basic Sciences, Loma Linda University School of Medicine, Loma Linda, CA 92354, USA; seanwilson@llu.edu

**Keywords:** kidney injury, vascular integrity, LPS, inflammation, calcium signaling

## Abstract

This study was undertaken to test two therapies for acute kidney injury (AKI) prevention, IGF-1, which is renal protective, and BTP-2, which is a calcium entry (SOCE) inhibitor. We utilized lipopolysaccharide (LPS) IP, as a systemic model of AKI and studied in five groups of animals. Three experiments showed that at 7 days: (1) LPS significantly reduced serum IGF-1 and intramuscular IGF-I in vivo gene therapy rescued this deficiency. (2) Next, at the 7-day time point, our combination therapy, compared to the untreated group, caused a significant increase in survival, which was noteworthy because all of the untreated animals died in 72 h. (3) The four pathways associated with inflammation, including (A) increase in cytosolic calcium, (B) elaboration of proinflammatory cytokines, (C) impairment of vascular integrity, and (D) cell injury, were adversely affected in renal tissue by LPS, using a sublethal dose of LPS. The expression of several genes was measured in each of the above pathways. The combined therapy of IGF-1 and BTP-2 caused a favorable gene expression response in all four pathways. Our current study was an AKI study, but these pathways are also involved in other types of severe inflammation, including sepsis, acute respiratory distress syndrome, and probably severe coronavirus infection.

## 1. Introduction

Acute kidney injury (AKI) is a common disease in hospitalized elderly patients and has high morbidity and mortality. AKI can be caused by renal ischemia, nephrotoxicity, or sepsis [1,2]. With respect to sepsis, globally, 1 in 5 deaths are due to sepsis-related problems, including AKI [3]. Despite considerable effort, there are no approved therapeutic agents for AKI as yet [4]. Our goal is to utilize an animal model of AKI in an attempt to develop a therapy that would lessen the renal injury, hasten the renal repair, and avoid the maladaptive repair leading to chronic renal failure [5,6]. The stage of AKI that is particularly detrimental to the patient is characterized by an increase in proinflammatory cytokines [7,8,9]. To treat this stage of AKI, we elected to utilize two therapeutic agents (IGF-1 and BTP-2). For our study, we used the AKI mouse model induced by parenteral LPS [10,11]. IGF-1 therapy was selected because there was a reduction in serum IGF-1 in our past studies of sepsis [12] and because IGF-1 is considered to be renal protective [13]. The mechanism for the decrease in serum IGF-1 in sepsis includes a depression of growth hormone receptor expression in the liver, which is a consequence of increased circulating inflammatory cytokines [12]. The liver is the main source of circulating IGF-1. Additionally, IGF-1 can cause the proliferation and differentiation of renal tubular cells, and it modulates immune cells to reduce proinflammatory cytokine production. With systemic LPS administration, there is usually a component of vascular injury [14]. IGF-1 has several functions that could counteract the effect of LPS to impair vascular function. IGF-1 increases endothelial adhesion molecules, increases proliferation of resident endothelial progenitor cells, increases the 1-α-hydroxylase that produces 1, 25 vitamin D (which is vascular protective), and is important for the activation of the sarcoplasmic endoplasmic reticulum calcium ATPase (SERCA), which is a calcium transporter important to the regulation of store-operated calcium entry (SOCE). SOCE in turn would decrease the high cytosolic calcium typical of inflammatory conditions, including LPS administration [15,16,17,18].

One of the primary early actions of LPS is to stimulate TLR-4, which leads to an increase in cytosolic calcium, which in turn mediates many of the adverse effects of LPS on cellular function [19,20]. Accordingly, the second agent that we selected, BTP-2 (YM–50483), is a potent inhibitor of SOCE via inhibition of the CRAC/Orai1 channel, which leads to a decrease in cytosolic calcium [21,22]. There are several potential inhibitors of SOCE [23], but we chose BTP-2 because it had been used successfully in experimental animals to suppress the immune system in severe inflammatory conditions [22,24,25]. Moreover, the immune-suppressive activity of BTP-2 is similar to cyclosporine, but cyclosporine is reportedly more toxic [26]. Thus, BTP-2 is potentially capable of blocking major detrimental pathways of LPS action, which lead to the production of proinflammatory cytokines.

Regarding the safety concerns, it has been reported that the BTP-2 calcium channel blocker does not cause cellular toxicity, even when used in higher concentrations [23]. However, for the use of BTP-2 in human clinical studies and translational advances, formal toxicity studies would be required.

## 2. Results

### 2.1. Strategy

**Figure 1:**
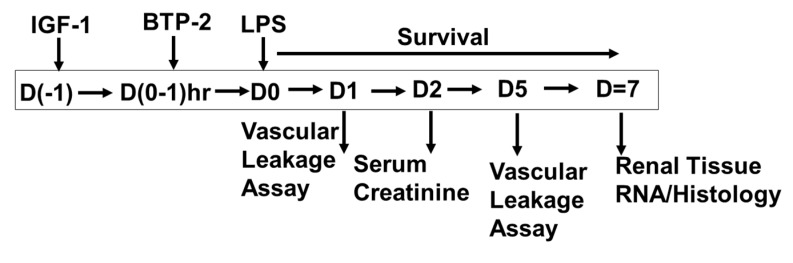
Experimental design for acute kidney injury (AKI) parameters such as survival, serum IGF-1 rescue and gene expression analysis in lipopolysaccharide (LPS) induced AKI animals treated with IGF-1 and BTP-2.

The objective of our studies was to develop a therapy regimen to prevent AKI. In this regard, three experiments were performed. (1) We sought to evaluate whether AKI inflammation would be associated with a decrease in serum IGF-1, as we had seen before with sepsis [12]. We found a marked decrease in serum IGF-1, and therefore, the next step was to rescue this deficiency, which we did by injecting skeletal muscle with an in vivo gene therapy approach, utilizing a Lentiviral vector engineered to express IGF-1 in skeletal muscle. In vivo gene therapy approach was utilized because this is a convenient approach for constant IGF-1 production. We reasoned that constant IGF-1 production would be necessary because the large molecular weight IGF binding protein 3 (IGFBP3) would be expected to be decreased because of liver growth receptor depression [27,28]. Therefore, the free IGF-1, being a small molecule, would be excreted in the urine. However, free IGF-1 in this context should be readily available to the site of injury in the kidney. The results in our current study are consistent with results previously published on IGF-1. However, the potential integration effects of the Lentiviral vector could have complemented or impaired our observed changes. The genome integration issue will be addressed in the future by utilizing clinically approved AAV- vectors, the use of which we have experience [29]. (2) In testing the efficacy of a new therapy for a lethal disease, the most critical test is survival. Therefore, we performed a survival test with a lethal dose of LPS (25 mg/kg body weight). We utilized animals treated with all components in combination therapy medications to get a comprehensive evaluation of our proposed therapeutic agents on survival. (3) We sought to evaluate the fundamental mechanisms associated with renal injury and repair. These mechanisms were studied by gene expression, histology, and, in the case of vascular integrity, Evans blue leakage [22]. Additionally, we studied gene expression for the processes of (1) intracellular calcium signaling, which is a major determinant of inflammatory cytokines, (2) vascular integrity, which is in part due to inflammatory cytokines, and (3) renal injury and repair, which is due to LPS toxicity and impaired vascularization (Figure 1).

### 2.2. Experiment 1

**Figure 2:**
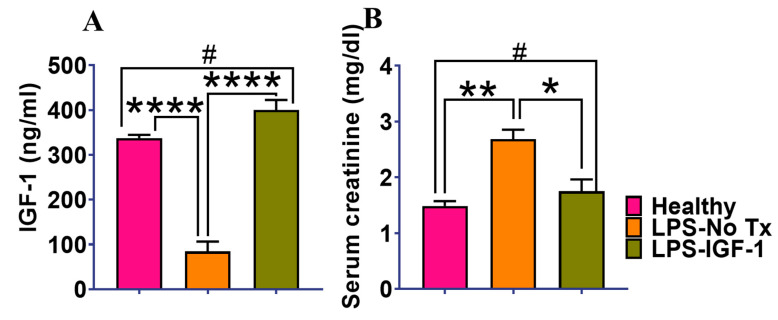
LPS induces a decrease in serum IGF-1 in B6 mice. LPS was administered IP on day 0, and the animals were sacrificed for measurements 7 days later. IGF-1 gene therapy was given one day before LPS administration. (**A**) shows the serum IGF-1 in the three groups of mice. (**B**) shows the correction of serum creatinine. IGF-1 gene therapy normalized the low serum IGF-1 and the high serum creatinine caused by the LPS administration. Data are mean ± SEM * *p* < 0.05, ** *p* < 0.01, **** *p* < 0.001, ^#^ not significant. 1-way ANOVA followed by a Dunnett’s multiple comparisons test (*n* = 3 in each group).

In this 7-day study, there was a marked drop in serum IGF-1 from 337.5 to 85 ng/mL (**** *p* < 0.001), as a consequence of the LPS treatment (20 mg/kg body weight). Intramuscular injection of the Lenti-IGF-1 vector rescued the serum IGF-1 deficiency and the elevated serum creatinine (Figure 2). We also evaluated the effects of IGF-1 + IGFBP3 because BP3 is the major IGF binding protein circulating in the blood [30]. However, we observed no superiority using the IGFBP3 in a survival study (data not shown), and therefore, we performed our studies with only in vivo IGF-1 gene therapy. Under normal conditions, IGF-1 circulates in a IGFBP3-bound form. However, under the conditions of LPS administration, IGFBP3 would be expected to be low [30]. Consequently, IGF-1 produced by muscle should circulate as free IGF-1, which would be readily accessible through the remaining intact vasculature to the injured kidney.

### 2.3. Experiment 2

**Figure 3:**
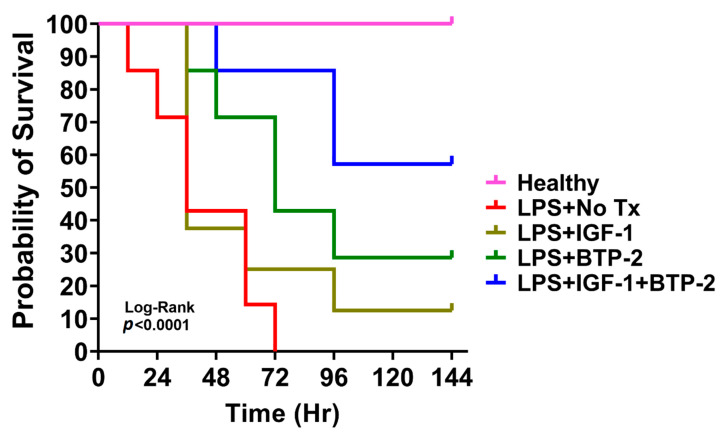
Overall survival of mice after the LPS injection and subsequent treatment. All of the animals except the healthy group were treated with 25 mg/kg of LPS IP. 144 h post-LPS injection, all of the surviving animals were sacrificed, and the experiment was terminated. Kaplan–Meier survival in response to LPS administration. Comparison of the trend of survival curve was done using the log-rank (Mantel–Cox) test (recommended), and **** *p* < 0.0001 (*n* = 7 in each group).

We examined whether IGF-1, BTP-2 monotherapies, and IGF-1 + BTP-2 combination therapy pretreatment protected mice from succumbing to death due to organ failure in AKI. Mice (7 per group) were injected (i.p) with a lethal dose of LPS (25 mg/kg body weight). One hour before the LPS injection, a group of mice received an i.p. injection of BTP-2 (16 mg/kg), and another group of mice received an i.m. injection of Lenti-IGF1, 24 h before LPS injection. A separate group of mice received a combination of IGF-1 + BTP-2 as pretreatment. The mice were monitored every 6 h. As shown in Figure 3, the mortality of mice injected with LPS reached 58% within 36 h and 100% within 72 h. The therapeutic regimens elicited varying degrees of protection. The mortality of LPS injected mice that received an IGF-1 pretreatment was 60% within 36 h, and 88% at the end of the experiment (144 h), whereas mortality in BTP-2 pretreatment group was 0% at 36 h, 30% at 48 h, and 72% at 144 h time point. The mortality rate was comparatively lower in the IGF-1 + BTP-2 group, which reached only 15% at 48 h, and at the end of the 144 h time point reached only up to 43%. Lower mortality and higher survival rates in the IGF-1+ BTP-2 group, when compared to No Tx and monotherapy groups, suggested that it offered a survival advantage to LPS-induced AKI mice. The overall survival rate was 0% in LPS-No Tx, 12.5% in IGF-1, 28% in BTP-2, and 57% in IGF-1 + BTP-2 at the end of the experiment (Figure 3). Meanwhile, no mortality was observed among healthy mice that were treated with PBS (Figure 3). Log rank analysis showed that there was a significant difference among the five groups of animals, indicating that IGF-1 + BTP-2 group survival of 57% was superior to the survival seen with the monotherapy groups.

### 2.4. Experiment 3

#### 2.4.1. Calcium Signaling

**Figure 4:**
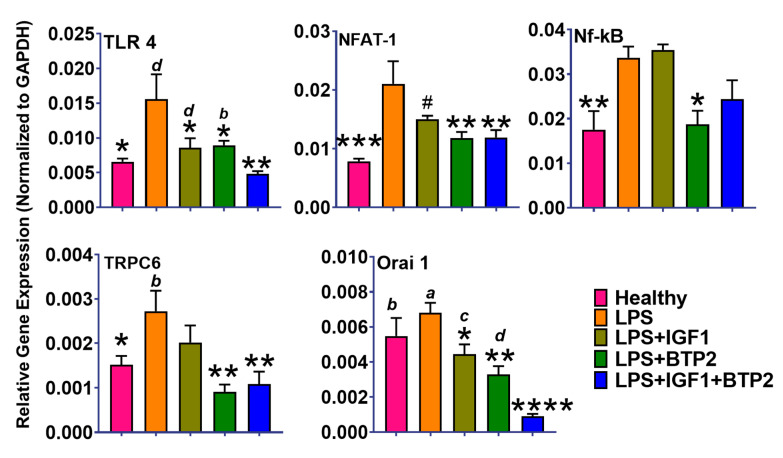
Favorable effects of therapies on LPS induced AKI expression of genes within the kidney related to the induction of calcium signaling. The target for BTP-2 is Orai1, which is decreased by this therapeutic agent as expected. See text for further interpretation. Data are mean ± SEM * *p* < 0.05, ** *p* < 0.01, *** *p* < 0.001, **** *p* < 0.0001, ^#^ not significant (LPS Vs. other groups), and *d*-*p* < 0.05, *c*-*p* < 0.01, *b*-*p* < 0.001, *a*-*p* < 0.0001 (LPS + IGF1 + BTP2 Vs. other groups), 1-way ANOVA followed by a Dunnett’s multiple comparisons test, (*n* = 4 in each group).

One of the earliest effects of LPS is to increase TLR-4 expression [20], which in turn increases Nfat, which is a transcription factor, classically known to be activated by increases in cytosolic calcium [31] (Figure 4). It is clear from earlier studies that the adverse action of LPS to produce AKI is determined heavily by aberrant calcium signaling [32,33]. The upstream events of Nfat have not been established for the kidney, but in lung microvasculature, the LPS decreases ER calcium stores via an IP3 receptor mechanism [34]. Emptying calcium stores would be expected to increase calcium influx from the plasma membrane (SOCE) via signaling by the endoplasmic reticulum calcium sensor Stim-1, which would promote an increase in orai1, a calcium specific calcium influx channel [35,36]. Accordingly, we expected to find an increase in Stim-1 and an increase in plasma membrane calcium-permeable ion channel Orai1 gene expression (or their analogs) as part of a mechanism to stimulate calcium influx through the plasma membrane. In this regard, we found a significant increase in Orai1 (Figure 4) but no change in Stim-1 (data not shown). The lack of a change in Stim-1 gene expression could have been related to the fact that we measured total kidney gene expression and not individual cell types thereof. Different cell types express variable plasma membrane-associated calcium signaling mechanisms [37,38]. Accordingly, in some cells, Stim stimulates calcium entry through a transient receptor potential (TRP) channel, instead of or in addition to, Orai1 [37]. We found a significant increase in TRPC6, as well as Orai1, in response to LPS (Figure 4), both of which would be expected to increase cytosolic calcium. Next, we decided to evaluate the effects of our therapy on these LPS induced cytosolic mechanistic calcium mechanisms.

BTP-2 is a specific inhibitor of Orai1/SOCE [22], and LPS increased Orai1 expression, whereas importantly, BTP-2 monotherapy decreased Orai1 expression to normal (Figure 4). Interestingly, IGF-1 monotherapy also decreased Orai1 expression by an unknown mechanism. Our combination therapy with IGF-1 +BTP-2 decreased Orai1 to below normal (Figure 4), an effect which could have contributed to our observed increase in survival with combination therapy (Figure 3). Next, we sought to determine downstream results from these improved effects of our therapy on cytosolic calcium dynamics. In this regard, it is established that elevated cytosolic calcium can lead to an increase in proinflammatory cytokines [39,40].

#### 2.4.2. Inflammatory Gene Expression

**Figure 5:**
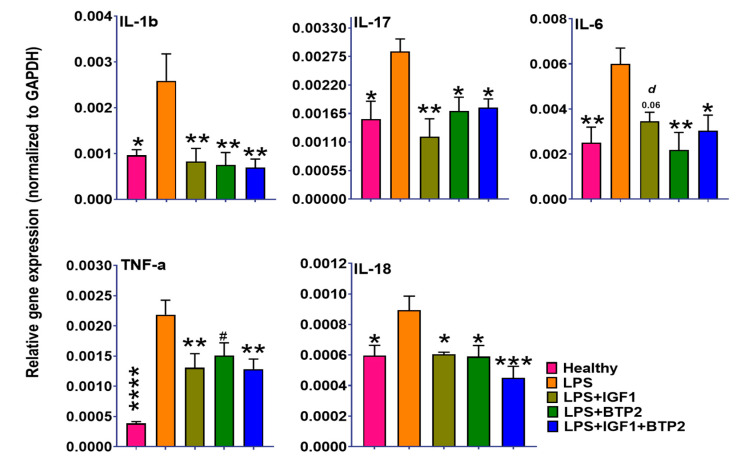
Favorable effects of IGF-1, BTP-2 monotherapies, and IGF-1 + BTP-2 combination therapies on LPS induced AKI associated major proinflammatory cytokines within the kidney. See text for interpretation. Data are mean ± SEM * *p* < 0.05, ** *p* < 0.01, *** *p* < 0.001, **** *p* < 0.0001, ^#^ not significant (LPS Vs. other groups) and *d*-*p* < 0.05, *c*-*p* < 0.01, *b*-*p* < 0.001, *a*-*p* < 0.0001 (LPS + IGF1 + BTP-2 Vs. other groups), 1-way ANOVA followed by a Dunnett’s multiple comparisons test, (*n* = 4 in each group).

Nfat and Nf-kB, which are activated by increases in cytosolic calcium, lead to production in proinflammatory cytokines [39,40], and both were increased in response to the LPS (Figure 4). Nf-kB increases the inflammatory cytokines, including IL-6, IL-1β, and IL-18, the latter being especially important because it synergizes with other cytokines [39,40] (Figure 5). Nfat increases proinflammatory cytokines (Th1, Th2, Th17) [41]. Th17 cells increase IL-17 (Figure 5), which can lead to maladaptive AKI repair [42]. All of the cytokines were decreased by IGF-1, BTP-2, and IGF-1 + BTP-2 combination therapies (Figure 5). Inflammatory cytokines are thought to directly contribute to the impaired vascular integrity seen with the LPS treatment [43]. Moreover, the cytokines also decrease the production of IGF-1 in the liver and possibly, locally at the inflamed sites in the kidney, which in turn could also have adverse effects on vascular integrity [43]. IL-18, which was also increased by LPS, is a product of the inflammasome, a calcium-dependent complex [44].

#### 2.4.3. Vascular Integrity

**Figure 6:**
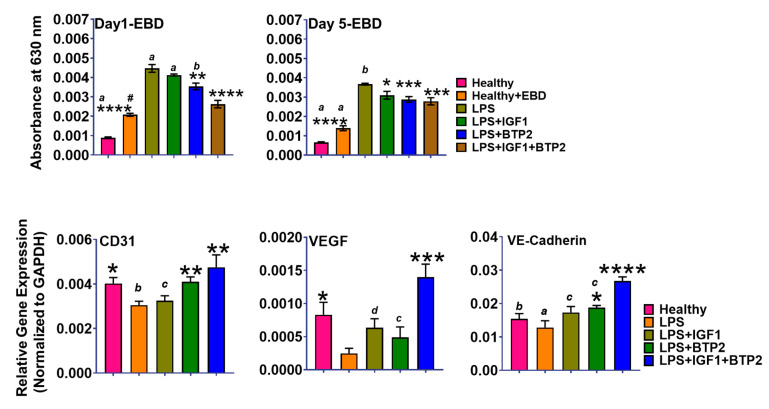
Favorable effects of therapies on LPS induced changes in vascular integrity in AKI mice. Evans blue dye measurements of vascular leakage in the kidney (Upper panel). CD31, VEGF, and VE-Cadherin, all angiogenic markers (lower panel). See text for interpretation. Data are mean ± SEM * *p* < 0.05, ** *p* < 0.01, *** *p* < 0.001, **** *p* < 0.0001 (LPS Vs. other groups) and *d*-*p* < 0.05, *c*-*p* < 0.01, *b*-*p* < 0.001, *a*-*p* < 0.0001 (LPS + IGF1 + BTP-2 Vs. other groups), 1-way ANOVA followed by a Dunnett’s multiple comparisons test, (*n* = 4 in each group).

Vascular leakage is one of the adverse effects of excess proinflammatory cytokine production. Vascular leakage is a serious consequence of systemic AKI; thus, we examined the extravasation of Evans blue dye for vascular leakage at day-1 and day-5 (Figure 6, upper panel). LPS injection induced a marked increase in vascular leakage, which was improved by IGF-1 and more so by BTP-2 treatment. Meanwhile, both agents showed a more significant reduction than either one alone at the day 1-time point. Importantly, the 5-day time point also showed significant decreases in vascular leakage by both agents alone and together.

Although the 5-day time point showed significant decreases in vascular leakage by the two agents together, the results were not any more favorable than results seen at the day-1 time point. However, gene expression of CD31 (endothelial cell adhesion molecules, (PECAM-1)), (Figure 6) was depressed by LPS, and increased by BTP-2 and the combination therapy. Consistent with the gene expression data was the finding by immunocytochemistry of an increase in CD31 staining in the sections from the combination therapy animals compared to the LPS group. Additionally, the VEGF expression was increased synergistically above normal in the dual therapy, suggestive of continuing repair. The marked decrease in the expression of P16, a tumor suppressor gene, was also consistent with increased cell proliferation, although it could have reflected changes in a variety of cell types since our samples contained renal tubular cells and immune cells, as well as endothelial cells (Figure S1). Perhaps influencing the only modest effect of our therapies on Evans blue dye leakage was the fact that the leakage study was done in 5 days. In the gene expression study, it was done at 7 days post-LPS administration.

#### 2.4.4. Renal Tubular Injury/Repair

**Figure 7:**
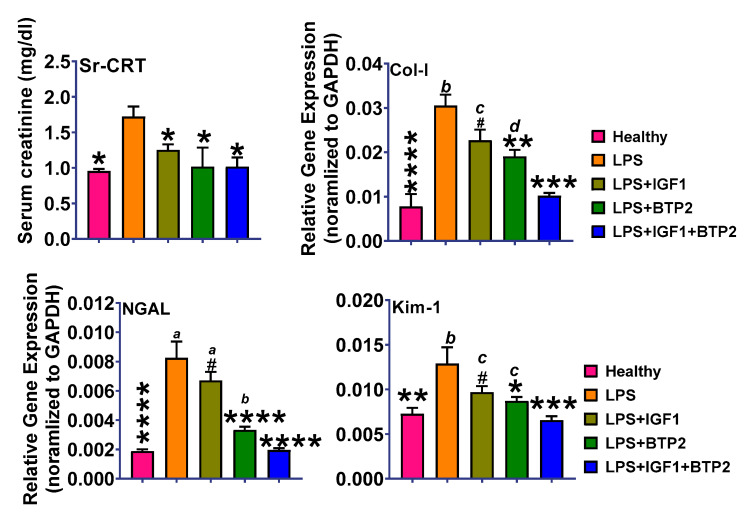
Favorable effects of IGF-1 and BTP-2 monotherapies and IGF-1 + BTP-2 combination therapy on LPS induced serum creatinine (Sr-CRT) and gene expression of the molecules reflecting kidney damage. See text for interpretation. Data are mean ± SEM * *p* < 0.05, ** *p* < 0.01, *** *p* < 0.001, **** *p* < 0.0001, ^#^ not significant (LPS Vs. other groups) and *d*-*p* < 0.05, *c*-*p* < 0.01, *b*-*p* < 0.001, *a*-*p* < 0.0001 (LPS + IGF1 + BTP-2 Vs. other groups), 1-way ANOVA followed by a Dunnett’s multiple comparisons test, (*n* = 4 in each group).

LPS injection induced a significant increase in serum creatinine, which was improved by IGF-1 and normalized by the combination therapy and by BTP-2 at 48 h (Figure 7). NGAL expression, which is more specific for renal function than serum creatinine, was markedly increased by LPS and improved by IGF-1 and BTP-2 and by the combination therapy (Figure 7). We then measured Kim-1 as its elevation is thought to reflect an increased risk of the development of maladaptive repair and interstitial fibrosis in kidney [5]. Kim-1 gene expression was also improved by single therapies and normalized by combination therapy. Furthermore, in our study, Kim-1 was normal at the 7-day time point, suggesting that this therapy might help to reduce the risk of ultimate maladaptation repair. Moreover, at 7 days, the expression of collagen type-1, a marker for tissue fibrosis, was markedly increased by LPS but normalized by combination therapy, again supporting the possibility that dual therapy decreases the risk of maladaptive repair. Unfortunately, our studies did not allow us to distinguish between damage and repair. For example, the improvement in NGAL could have been due to less damage or increase in repair.

#### 2.4.5. Kidney Histopathology

**Figure 8:**
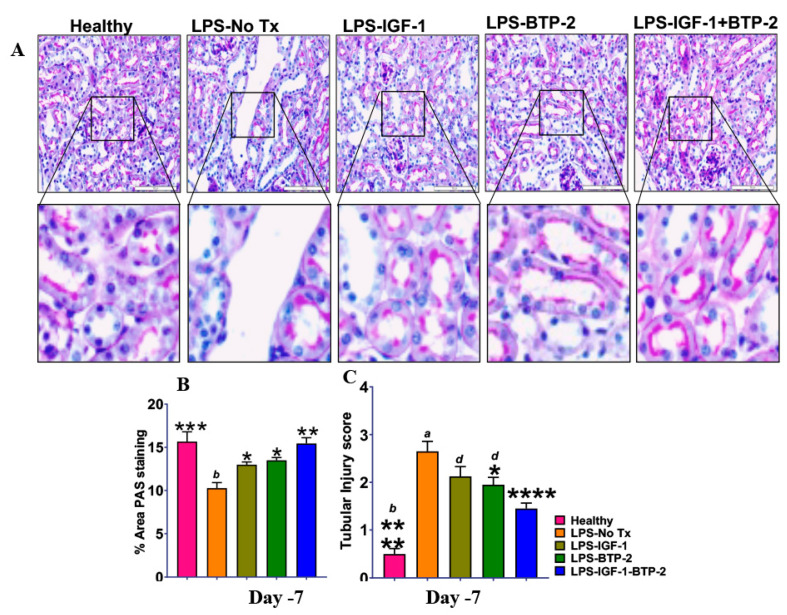
Effect of therapies on LPS induced acute tubular injury. (**A**) Periodic acid–Schiff (PAS) staining on day 7 of kidney injury (original magnification 40×; Scale bar, 100 µm). (**B**) % Area pf PAS staining. (**C**) Acute tubular injury score of mouse kidneys. Data are mean ± SEM * *p* < 0.05, ** *p* < 0.01, *** *p* < 0.001, **** *p* < 0.0001 (* LPS Vs. other groups) and *d*-*p* < 0.05, *c*-*p* < 0.01, *b*-*p* < 0.001, *a*-*p* < 0.0001 (LPS + IGF1 + BTP-2 Vs. other groups), 1-way ANOVA followed by a Dunnett’s multiple comparisons test, and unpaired *t*-test.

Quantitative measurements of periodic acid–Schiff (PAS) staining of the kidney tubule brush border were performed to evaluate kidney damage (Figure 8). LPS caused a decrease in staining, and there were significant improvements by single therapy and normalization by combination therapy. The tubular injury score was consistent with the results of the PAS staining. There was a marked increase in the score in the LPS group and decreases in the score in single therapies, and particularly, the combination therapy. However, in contrast to the PAS staining, which was normalized in the combination therapy group, the tubular injury score was still elevated above the healthy group. Therefore, despite the normalization of AKI injury gene expression and PAS staining, there was statistically significant evidence of residual renal damage by the histopathology score.

**Figure 9:**
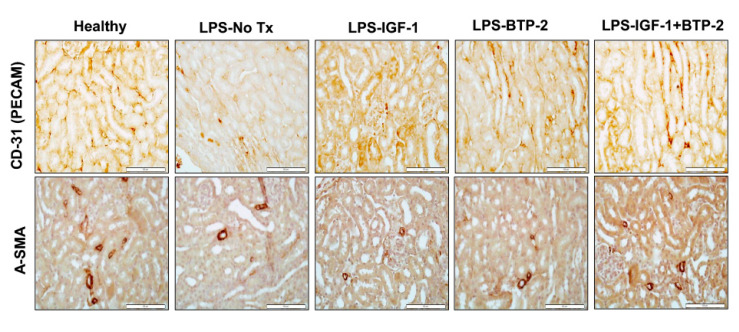
Effects of LPS in therapies on renal vascular integrity histologic parameters. (Upper panel) CD31 (PECAM) histology staining of renal sections, (Lower panel) a-SMA histology staining of renal sections (original magnification 40×; Scale bar, 100 µm).

LPS administration caused a marked decrease in PECAM (CD-31) staining, a change which was improved by IGF-1 therapy and also by BTP-2 therapy. In the combination therapy group, staining was improved over single therapy results but perhaps slightly decreased from normal (Figure 9). Similarly, LPS administration caused a decrease in α-SMA staining, a change that was improved by IGF-1 therapy and also by BTP-2 therapy. In the combination therapy group, the staining was improved over single therapy groups and appeared to be equivalent to normal. Both PECAM (CD-31) and α-SMA staining are parameters related to vascular integrity. Therefore, our results showed substantial improvements with therapy in vascular gene expression and vascular staining parameters, but a substantial residual impairment in vascular leakage.

## 3. Discussion

The most important observation of the current study is the demonstration, in a mouse model of systemic AKI, that successful therapeutic intervention is feasible. Accordingly, we found that the combination therapy of BTP-2+ IGF-1 caused a 57% survival to a lethal dose of LPS and that the combination therapy was significantly better than either monotherapy compared to the untreated group. Our survival results from this initial study are exciting. In support of the survival data, we found favorable responses in our mechanistic studies of gene expression and histopathology evaluations of renal tubular and renal vascular status.

After obtaining the successful survival data, the next step was to evaluate the mechanism for the beneficial combination therapy. Mechanistic studies were considered to be critical for our long-term goal to improve survival, which is currently at 57%.

**Figure 10:**
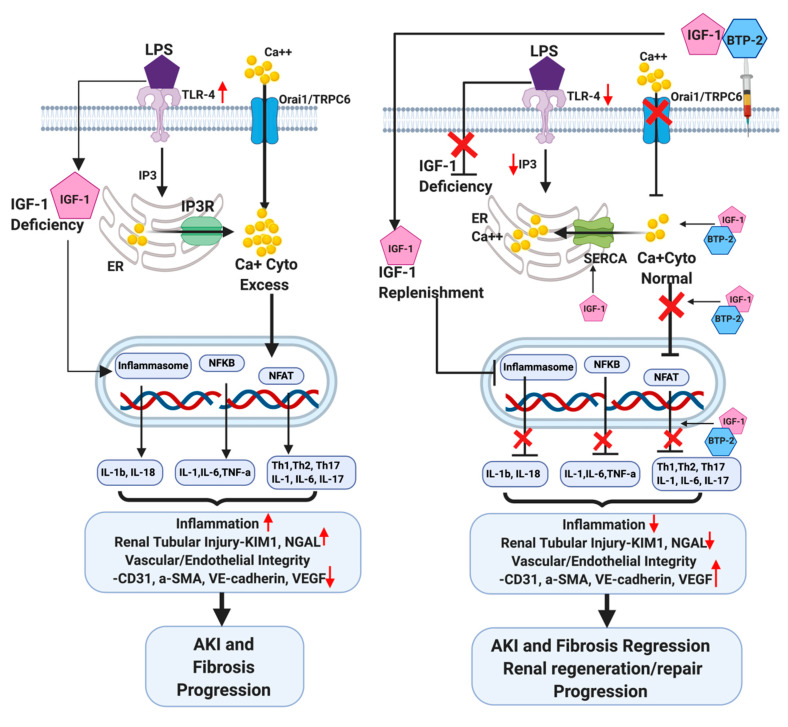
Schematic molecular pathway responses to the induction of AKI by LPS and the preventative therapeutic counteractions by IGF-1 and BTP-2. The left panel shows the effects of LPS, while the right panel shows the effects of combination therapy, IGF-1 + BTP-2. LPS initiates the destructive process by activating TLR-4, which leads to an enhancement of IP3 receptor activity in the ER, which in turn enhances the calcium reflux from the ER to the cytosol. The depletion of ER calcium activates plasma membrane calcium influx, via Stim, through Orai1/TRPC6 channels. The consequent increase in cytosol calcium propels the pathway that leads to inflammation and injury, as described in the text. On the right panel, the red X indicates the sites of action of combination therapy (IGF-1 + BTP-2), which had positive effects on all of the adverse system changes caused by LPS administration. The action of BTP-2 is to specifically inhibit Orai1 and TRPC6, which counteracts the effect of LPS on calcium influx into the cytosol. The function of BTP-2 to inhibit the calcium channel regulator is complemented by IGF-1, which acts on SERCA to increase calcium influx into the ER from the cytosol. The consequent decrease in cytosolic calcium counteracts much of the adverse actions of LPS, as described in the text.

Regarding mechanisms, the TLR-4 gene was markedly increased by the LPS administration and normalized in 7-days by IGF-1 + BTP-2 combination therapy. One of the most fundamental actions of TLR-4 activation is to increase cytosolic calcium [19,20]. TLR-4 activity increases the synthesis of proinflammatory cytokines in various cells at the site of inflammation [19,20], which in excess could harm renal tubular function directly and through the corresponding decrease in vascular integrity such as we observed. There has been a thorough analysis of the mechanisms whereby LPS increases cytosolic calcium in the kidney [32,45]. Based on our data and in the literature, we developed a model to facilitate an understanding of the therapeutic actions of our dual therapy (Figure 10). We have chosen as an early effect LPS, an increase in the IP3 receptor-induced calcium release from the ER into the cytosol, which has an established effect in LPS on lung microcapillaries [34].

The next step in our model is an increase in one or more STIM analogs, which is known to occur in response to the depletion of calcium from the ER stores [46]. STIM is well known to activate Orai1, which is the core of the CRAC calcium channel that provides for calcium entry across the plasma membrane following depletion of the ER calcium store. Similarly, STIM may also activate TRPC6, which we found to occur in response to the LPS that also increases cytosolic calcium through influx across the plasma membrane [20,47]. We did not find any change in STIM-1 expression, though this does not discount the potential that one of the other STIM analogs were upregulated or because we did not study a specific cell type, but rather a heterogeneous population of kidney cells. Moreover, the interactions among SOCE components and the regulation of cytosolic calcium is complex and not fully understood [35]. Moreover, it is quite possible that by 7 days, any increment in STIM-1 had become normal in response to therapy. In any case, in support of our model, we found a slight increase in Orai1 in response to LPS and a marked decrease in response to BTP-2 therapy. It is noteworthy that this marked decrease in Orai1 occurred a full 7 days after a single administration of BTP-2, raising the possibility of a prolonged biological half-life. Although we did not measure cytosolic calcium in these studies, our finding of an increase in Nfat in response to LPS strongly supports our conclusion that there was a sustained increase in cytosolic calcium.

Our data are consistent with the conclusion that BTP-2 decreases calcium influx into the cytosol by both TrpC6 and the CRAC channels. There is an additional potential mechanism involved in the reduction of cytosolic calcium using our dual therapy. With respect to the effect of LPS to promote ER calcium release into the cytoplasm [34], IGF-1 is known to stimulate SERCA in muscle tissue, which is an ATPase functioning to transport calcium from the cytosol into the ER [48]. If this were applicable to our system, as illustrated in Figure 10, it would indicate that not only BTP-2 but also IGF-1 would act to decrease elevated cytosolic calcium caused by LPS administration.

One finding of potential clinical significance about Orai1 positive cells is that they have been shown to be responsible for the chronic maladaptive action to AKI that eventually leads to interstitial fibrosis and chronic renal disease. In this regard, our combination therapy decreased expression of Orai1 to below the normal level, and this was associated with a rescue of the increase in type I collagen renal tissue at 7 days (Figure 7).

The only major process that was not substantially improved in 5 to 7 days of dual therapy was vascular integrity, as evidenced by vascular leakage, for which there were several potential applicable causes in our study. (1) Increased proinflammatory cytokines expression, such as IL-1 and TNF-α, which we observed in the present study and which can lead to vascular leakage (Figure 5) [43]. (2) LPS induces an increase in cytosolic calcium, which leads to an increase in PKC-α, which in turn disassembles VE-Cadherin, a major endothelial adhesion molecule [49,50]. In our study, we found a marked decrease in expression of VE-Cadherin (Figure 6) with LPS administration and a favorable overcorrection with the combination therapy. The overcorrection suggests that the process of vascular repair was not complete. (3) LPS decreased α-SMA expression (which is a marker for Pericytes) and our combination therapy improved α-SMA staining as determined by immunocytochemistry. A loss of pericytes is associated with vascular leakage [14]. (4) Decreased 1,25-dihydroxyvitamin D synthesis (the barrier hormone) could have occurred because it requires adequate IGF-1 levels and because IGF-1 is decreased in response to sepsis [12] and in the current study as well. (5) In our study, combination therapy increased VEGF expression above normal in the kidney (Figure 6). We speculate that the increase in VEGF was a compensatory effort to repair the vascular injury. However, it is noteworthy that VEGF alone can cause vascular leakage, apparently by causing the formation of new capillaries, which typically exhibit increased permeability [51]. This raises the possibility that increasing the length of the therapy would afford further improvements. Meanwhile, our IGF-1 treatment is also a potential mechanism for the increase in VEGF gene expression [52].

Because the impairment in vascular permeability at 7 days was greater than the architectural abnormality of histologic tubular injury, perhaps the defect in vascular integrity in response to LPS after 7 days of combination therapy is due to both architectural as well as functional vascular impairment.

With respect to tubular cell injury, LPS caused marked increases in both NGAL and Kim-1, which are markers of renal tubular cell injury [53,54]. Importantly, our combination therapy normalized the increase in NGAL expression and markedly reduced Kim-1 expression. Despite these favorable changes in gene expression, our histology studies indicate that the injury score was higher than normal. However, this score did considerably improve with combination therapy. Histologic studies indicate architectural abnormalities in renal tubules at 7 days.

In conclusion, we suggest that our combination therapy shows potentially favorable progress toward the development of clinical therapy for AKI. Our results emphasize the importance of vascular dysfunction as a process that needs further assessment, and our results, together with the results of others, highlight the importance of dysfunctional calcium signaling with AKI. This process can be manipulated therapeutically to improve AKI.

In general terms, the importance of our dual therapy potentially extends beyond AKI in that other acute inflammatory syndromes, such as acute pulmonary injury, sepsis, coronavirus infection, and major/trauma surgery, are potentially amenable to our combination therapy. Relevant preclinical follow-up studies will include an attempt to optimize dose and time responses, improve renal vascular integrity, and evaluate our optimized therapy on AKI models other than sepsis [55].

## 4. Material and Methods

### 4.1. Experimental Design

There were three experiments using B6 mice between 5–8 weeks of age. First, based on our experience [12], we evaluated the effect of LPS to induce IGF-1 deficiency, and if so, to rescue that deficiency using in vivo IGF-1 gene therapy. The second experiment tested the effect of our therapies on survival at 7 days. There were five groups of animals, (1) healthy, (2) LPS untreated (LPS-No Tx), (3) IGF-1 in vivo gene therapy one day before LPS injection (LPS + IGF-1), (4) BTP-2 16 mg/kg IP at the start of the study, and the (5) combined therapy with IGF-1 and BTP-2 (LPS-IGF-1 + BTP-2), (Figure 2). In the third experiment, we sought to obtain mechanistic molecular pathway data and histopathology data. In the survival experiment, we used a lethal dose of LPS (25 mg/kg) and all of the untreated animals died within 72 h. Therefore, in the third experiment, a sublethal dose of LPS (20 mg/kg) was used.

### 4.2. Animals

Female C57/BL6 mice were purchased from The Jackson Laboratory (Bar Harbor, ME, USA). All mice were used at ages 5–8 weeks. The investigators adhered to the Animal Welfare Act regulations and other Federal statutes relating to animals and experiments involving animals and the principles set forth in the current version of the Guide for Care and Use of Laboratory Animals, National Research Council. All experiments were performed according to protocols approved by the Institutional Animal Care and Use Committee at the Loma Linda University on 18 December 2018.

### 4.3. LPS Induced Sepsis and Subsequent AKI

Female C57BL/6 mice received a sublethal or lethal dose of LPS from *E. coli* (0127: B8 strain, Sigma Aldrich, St. Louis, MO, USA) in a concentration of 20 or 25 mg/kg body weight administrated in sterile PBS by intraperitoneal injection.

### 4.4. Treatment

In a preventive, therapeutic strategy, some of the mice received a dose of ~3.0 × 10^6^ TU in a 20 μL volume of an intramuscular injection of Lenti-IGF-1 (Supplementary Method S1) 24 h before LPS injection; whereas, the calcium signaling blocker BTP-2 (16 mg/kg, Cayman Chemical, Ann Arbor, MI, USA) was administrated 1 h before the LPS injection. Some of the animals received a combination of Lenti-IGF-1 and BTP-2. The control group mice were injected with an equal volume of PBS. 

### 4.5. Renal Function Assessment

Blood samples were collected through a facial vein puncture at 48 h. The serum was separated at 3000 rpm for 5 min at 4 °C. Serum creatinine (CREA) was detected using a Creatinine Assay Kit (Cayman Chemicals, Ann Arbor, MI, USA).

### 4.6. Measurements of mRNA Expression in the Kidney

For the measurement of the mRNA expressions of whole kidney tissue, we performed real-time PCR. The sequences of the primer and gene database numbers are listed in Table S1. The relative amount of mRNA was calculated using the comparative *C*_t_ (*∆∆C*_t_) method. All specific amplicons were normalized against GAPDH [29].

### 4.7. Vascular Leakage Permeability Assay

Evans blue dye (EBD) was dissolved in a 0.9% saline solution at a concentration of 5 mg/mL and injected to the mouse tail vein (50 mg/kg, i.v.). After 30 min, the kidneys were harvested, dried, and weighed. Dried tissues were soaked in 3 mL of formamide and homogenized using a homogenizer followed by incubation at 60 °C for 18 h. The homogenized tissues were centrifuged at 12,000× *g* for 30 min. The absorbance of the supernatants was measured at 620 and 740 nm using a dual-wave ELISA plate reader [56].

### 4.8. Renal Histology

After sacrificing the mice, one part of the kidney was immediately cut, fixed in a 10% neutral buffered formalin solution, embedded in paraffin, and used for histopathological examination. Then, 10-micrometer-thick sections were cut, deparaffinized, and hydrated. All renal serial sections were incubated at 4 °C overnight with one of two antibodies; rabbit polyclonal antibody against PECAM (CD31) (Santa Cruz, MA) or rabbit polyclonal antibody against α-SMA (Abcam, Cambridge, MA, USA). After washing with TBST, biotinylated goat anti-rabbit IgG (1:200, Vector Labs, Burlingame, CA, USA) were applied to the sections for 30 min at room temperature. Sections were then incubated with Streptavidin-HRP (Vector Labs, Burlingame, CA, USA) for 30 min at room temperature. Diaminobenzidine (DAB; Vector Labs, Burlingame, CA, USA) was used as the chromogen and hematoxylin as the counterstain.

One part of the kidney was stained with periodic acid-Schiff (PAS) stain. The PAS stained sections were examined for renal morphology in an automated fashion by quantification of a glycogen specific color in the kidney tissue using ImageJ (National Institutes of Health, Bethesda, MD, USA). A minimum of 10 fields for each kidney slide were examined and scored for pathological injury. A score from 0 to 4 was given for pathological assessment: 0, normal histology; 1, mild injury, 5% to 25% of tubules showed pathological damage; 2, moderate injury, 25% to 50% of tubules showed pathological damage; 3, severe injury, 50% to 75% showed pathological damage; and 4, almost all tubules in field of view were damaged. The average histological score for each sample was calculated. The images were captured with an Olympus BX51 microscope, 40× magnification. (Olympus, Center Valley, PA, USA) [57].

### 4.9. Statistical Analysis

Statistical analyses were performed with GraphPad software (Prism 5.02). Kaplan–Meier survival studies were analyzed using the log-rank test and the Gehan–Breslow–Wilcoxon test. The quantitative analyses such as Q-PCR data are reported as the mean ± SEM, and they were analyzed using 1- or 2-way ANOVA followed by a Dunnett’s multiple comparisons test or a Bonferroni post hoc analysis as appropriate, or an unpaired *t*-test. Evaluation of the histopathology preparations was blind, and specimen identity was revealed only on completion of analysis. A *p*-value of <0.05 was considered to be statistically significant.

## Supplementary Materials

Supplementary materials can be found at https://www.mdpi.com/1422-0067/21/11/4095/s1. **Figure S1**. Positive effects of therapies on LPS induced changes P16 gene expression. See text for interpretation. Data are mean ± SEM * *p* < 0.05, ** *p* < 0.01, *** *p* < 0.001, **** *p* < 0.0001 (* LPS Vs. other groups) and d-*p* < 0.05, c-*p* < 0.01, b-*p* < 0.001, a-*p* < 0.0001 (LPS + IGF1 + BTP2 Vs. other groups) (*n* = 4 in each group); **Table S1**. Primer List.
