# Supplementary material for: IGF-1 Deficiency Rescue and Intracellular Calcium Blockade Improves Survival and Corresponding Mechanisms in a Mouse Model of Acute Kidney Injury
Source: Int J Mol Sci. 2020 Jun 8;21(11):4095. doi: 10.3390/ijms21114095 (PMC7312627; doi:10.3390/ijms21114095)
Supplement: Supplementary file 1 [file ijms-21-04095-s001.pdf]

Supplementary information:

Supplementary Figure

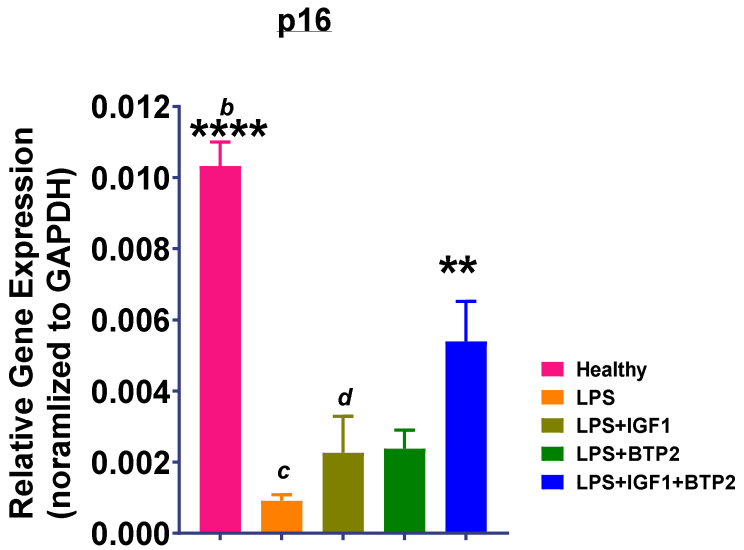

Figure 1. Positive effects of therapies on LPS induced changes on P16 gene expression. See text for interpretation. Data are mean ± SEM * $p < 0.05$, ** $p < 0.01$, *** $p < 0.001$, **** $p < 0.0001$(* LPS Vs other groups) and ***d***-$p < 0.05$, ***c***-$p < 0.01$, ***b***-$p < 0.001$, ***a***-$p < 0.0001$ (LPS+IGF1+BTP2 Vs other groups) ($n = 4$ in each group).

Supplementary Table

Table S1 Primer List		
**Gene**	**Forward**	**Reverse**
TLR-4	GCCTTTCAGGGAATTAAGCTCC	GATCAACCGATGGACGTGTAAA
NFATC1	GGAGAGTCCGAGAATCGAGAT	TTGCAGCTAGGAAGTACGTCT
NFKB	ATGGCAGACGATGATCCCTAC	TGTTGACAGTGGTATTTCTGGTG
TRPC6	GCTTCCGGGGTAATGAAAACA	GTATGCTGGTCCTCGATTAGC
ORAI 1	CTCAACTCGGTCAAAGAGTCAC	CACGACCTCTGCTAGGAAAAG
IL-1B	GAAATGCCACCTTTTGACAGTG	TGGATGCTCTCATCAGGACAG
IL-17	GGCCCTCAGACTACCTCAAC	TCTCGACCCTGAAAGTGAAGG
IL-6	CTGCAAGAGACTTCCATCCAG	AGTGGTATAGACAGGTCTGTTGG
TNF-A	CCTGTAGCCCACGTCGTAG	GGGAGTAGACAAGGTACAACCC
IL-18	GACTCTTGCGTCAACTTCAAGG	CAGGCTGTCTTTTGTCAACGA
CD-31	CTGCCAGTCCGAAAATGGAAC	CTTCATCCACCGGGGCTATC
VEGF	GCACATAGAGAGAATGAGCTTCC	CTCCGCTCTGAACAAGGCT
VE-CADHERIN	GTCGATGCTAACACAGGGAATG	AATACCTGGTGCGAAAACACA
NGAL	GGGAAATATGCACAGGTATCCTC	CATGGCGAACTGGTTGTAGTC
KIM-1	ACATATCGTGGAATCACAACGAC	ACTGCTCTTCTGATAGGTGACA
COL-I	TAAGGGTCCCCAATGGTGAGA	GGGTCCCTCGACTCCTACAT
P-16	GCTCAACTACGGTGCAGATTC	GCACGATGTCTTGATGTCCC
GAPDH	TGGCCTTCCGTGTTCCTAC	GAGTTGCTGTTGAAGTCGCA

Notes: TLR-4: Toll-like receptor 4, NFATC1: Nuclear factor of activated T cells 1; NFKB: Nuclear factor kappa B; TRPC6: Transient receptor potential cation channel subfamily C member 6, ORAI-1: ORAI calcium release-activated calcium modulator 1, IL-1B: Interleukin 1 beta, IL-17:Interleukin 17; IL-6: Interleukin-6; TNF-α :Tumor Necrosis Factor -α, IL-18: Interleukin 18, CD-31: Platelet/endothelial cell adhesion molecule 1, VEGF: Vascular endothelial growth factor, VE-Cadherin: Endothelial-Specific Cadherin, NGAL:Lipocalin 2; Kim-1: Kidney Injury Molecule-1, Col1: Collagen type I, p16: cyclin dependent kinase inhibitor 2A, GAPDH: Glyceraldehyde-3-Phosphate Dehydrogenase

Supplementary methods

Methods S1 IGF-1 Lentivirus

The cDNAs for mouse IGF-1 were purchased from GeneCopoeia. For the production of incompetent lentiviruses, we used the standard protocol. Vector stocks of vesicular stomatitis virus glycoprotein (VSV-G)–pseudotype lentiviral vectors were prepared by calcium phosphate–mediated 3-plasmid transfection of 293T cells. Briefly, 27 μg transfer vector construct, 17.5 μg second-generation gag-pol packaging construct pCMV.R8.74, and 9.5μg VSV-G expression construct pMD.G were used for transfection of 12 x 10^6 293T cells overnight in 25 mL Dulbecco modified Eagle medium (DMEM) with 10% heat-inactivated fetal bovine serum. The cells were treated with 10 mM sodium butyrate during the first of three 12-hour vector supernatant collections. The supernatant was filtered through 0.45-μm-pore-size filters (Nalgene Nunc) and concentrated 100-fold by ultracentrifugation before freezing and storing at -80° C.
